# Effect of nighttime bedroom temperature on heart rate variability in older adults: an observational study

**DOI:** 10.1186/s12916-025-04513-0

**Published:** 2025-12-29

**Authors:** Fergus K. O’Connor, Aaron J. E. Bach, Connor Forbes, Shannon Rutherford, Sebastian Binnewies, Surendran Sabapathy, Norman R. Morris

**Affiliations:** 1 School of Allied Health, Sport and Social Work, Griffith University, 1 Parklands Dr, Southport, QLD 4215 Australia; 2 Australian Centre for Precision Health and Technology, Griffith University, Gold Coast, QLD Australia; 3 Griffith Institute for Human and Environmental Resilience, Griffith University, Gold Coast, QLD Australia; 4 School of Information and Communication Technology, Griffith University, Gold Coast, QLD Australia; 5 School of Medicine and Dentistry, Griffith University, Gold Coast, QLD Australia; 6 Allied Health Research Collaborative, The Prince Charles Hospital, Metro North Hospital and Health Service, Brisbane, QLD Australia

**Keywords:** Indoor temperature, Heat stress, Thermoregulation, Autonomic stress

## Abstract

**Background:**

Climate change is increasing the frequency of hot nights, which may contribute to cardiovascular morbidity and mortality by impairing sleep and autonomic recovery. Despite World Health Organization guidelines for maximum daytime indoor temperatures (26 °C, 79 °F), there are no equivalent recommendations for nighttime conditions. We investigated the impact of nocturnal bedroom temperature on heart rate and heart rate variability (HRV) in free-living older adults.

**Methods:**

In this observational study, 47 community-dwelling adults aged ≥ 65 years in southeast Queensland, Australia, were monitored across one summer (December 2024–March 2025). Wearable devices recorded heart rate and HRV during nighttime periods of sleep between the hours of 9 PM–7 AM, while in-home sensors continuously measured bedroom temperature. The primary outcome was the natural logarithm of the root mean square of successive differences (lnRMSSD). Secondary outcomes were log-transformed frequency-domain HRV indices (high frequency: lnHF, low frequency: lnLF, low to high frequency ratio: lnLF:HF) and heart rate. Generalised mixed effects models analysed associations between wearable derived outcomes and temperature categories (< 24 °C [79 °F], 24–26 °C [75–79 °F], 26–28 °C [79–82 °F], 28–32 °C [82–90 °F]). Clinically relevant thresholds were defined as ≥ 1.5 standard deviation change in HRV or ≥ 5 beats·min⁻^1^ change in heart rate.

**Results:**

Across 14,179 valid nighttime hours, median bedroom temperature was 25.9 °C (Q1, Q3; 24.6, 26.9). Compared with < 24 °C (79 °F), nighttime bedroom temperatures of 24–26 °C (75–79 °F; odds ratio: 1.4; 95% confidence interval [1.2, 1.6], *P* < 0.001), 26–28 °C (79–82 °F; 2.0 [1.8–2.3]) and 28–32 °C (82–90 °F; 2.9 [2.5–3.4]) were associated with greater odds of clinically relevant reductions in lnRMSSD (*P* < 0.001). Higher temperatures were also linked to reduced lnHF and lnLF, increased ln(LF:HF), and elevated heart rate (all *P* < 0.001).

**Conclusions:**

Nocturnal bedroom temperatures above 24 °C (79 °F) were associated with greater likelihood of autonomic disruption and increased heart rate in older adults, consistent with a shift toward sympathetic dominance and heightened physiological stress, with greater effects observed as temperature increased. These findings provide real-world physiological evidence supporting the development of nighttime indoor temperature guidelines to protect vulnerable populations in a warming climate.

**Supplementary Information:**

The online version contains supplementary material available at 10.1186/s12916-025-04513-0.

## Background

The impact of climate change–driven increases in ambient temperature on morbidity and mortality is well established, with older adults among the most at-risk [1–3]. While a vast body of literature has documented the adverse health effects of extreme daytime heat [4], comparatively less is known about the consequences of elevated nighttime temperatures [1]. Emerging evidence suggests that hot nights may exert an independent and cumulative physiological burden, by impairing sleep quality and limiting overnight recovery, contributing to increased morbidity and mortality, particularly in vulnerable populations [1]. Importantly, climate projections forecast a shift wherein, by the year 2100, a larger proportion of heat-related deaths will result from hot nights rather than hot days [5]. Despite this, while the World Health Organization provides recommendations for maximum daytime indoor temperature limits to protect human health (26 °C, 79 °F) [6], there is a distinct lack of guidance for the maintenance of indoor nighttime temperatures.

Alongside sleep disturbances, the health implications of hot nights are hypothesised to arise from increased oxidative vascular damage and systemic inflammatory responses [1], which may disrupt autonomic function and increase heat vulnerability. Although direct monitoring of these responses is challenging outside of laboratory settings, advancements in wearable technology have enabled continuous physiological monitoring in free-living environments. Indeed, these systemic stress responses can be indirectly captured using wearable-derived heart rate and heart rate variability measures [7], which serve as surrogate markers of autonomic regulation. Laboratory studies have confirmed the influence of prolonged heat exposure on heart rate variability measures in older adults [8], where lower heart rate variability indicated worsened autonomic regulation and higher cardiovascular risk [9]. However, little information currently exists detailing the influence of nocturnal bedroom temperature of heart rate variability responses in free-living older adults.

Alongside a lack of real-world physiological data to substantiate proposed mechanisms and heightened risk profiles, the misalignment between epidemiology, climate-projections, and current guidance poses challenges for policymakers and public health officials aiming to mitigate the health risks associated with elevated indoor temperatures, especially during nighttime hours. This regulatory gap underscores a critical limitation in current heat-health frameworks, which fail to adequately address the unique physiological demands of nighttime thermal exposures. As hot nights become increasingly prevalent, the absence of specific nocturnal indoor temperature guidelines may leave vulnerable populations at heightened risk of adverse health outcomes. To address this knowledge gap and provide preliminary data for the development of indoor nighttime temperature limits, we conducted continuous in-home monitoring of nighttime bedroom temperatures and heart rate and heart rate variability responses in free-living older adults throughout an entire Australian summer season.

## Methods

Following approval of the Human Research Ethics Committee of Griffith University (GUREF2024/642) adults aged ≥ 65 years from southeast Queensland, Australia, volunteered and provided written informed consent for the observational study, which ran from December 1st, 2024, to March 17th, 2025.

Participants were recruited between September and October 2024. Potential participants were eligible for this observational study if they were ≥ 65 years old, English speaking, willing to wear a heart rate and activity monitor on their wrist for the duration of the summer-long observational period, and were able to provide written, informed consent. Participants were recruited from southeast Queensland, Australia, which has a humid subtropical climate with year-round warm to hot temperatures (Köppen climate classification: Cfa). Table 1 details the full inclusion/exclusion criteria.

**Table 1:** Trial eligibility criteria

Inclusion criteria
• Male or female adults^a^
• Aged ≥ 65 years^a^
• Non-smoking^a^
• English speaking
• Ability to provide informed consent
• Meeting the basic criteria of age and location and availability during study period (minimum of 60 days of the data collection period)
• Have an iPhone 6 or Android equivalent, or higher
• Willing and able to wear a fitness data tracker throughout the duration of the study period
Exclusion criteria
• Physical restriction (e.g. due to disease: intermittent claudication, renal impairment, active proliferative retinopathy, unstable cardiac or pulmonary disease, disabling stroke, and severe arthritis)^a^
• Use of or changes in medication judged by the patient or investigators to make participation in this study inadvisable^a^

^a^Participant sex, age, smoking status, physical restrictions, and medication use evaluated through participant self-report

### General procedures

#### Measurement tools

##### Heart rate and heart rate variability

Participants were supplied with wearable physiological monitoring devices (Inspire3, Fitbit, Google, USA) prior to the observational period and were instructed to wear the device on their non-dominant wrist for the duration of the trial period, spanning the Australian summer.

Heart rate was continuously monitored using photoplethysmography (PPG), a non-invasive optical technique that estimates blood volume changes in the microvascular bed of tissue [10]. Specifically, heart rate was derived from pulse wave signals detected at the wrist, reflecting changes in capillary blood flow. Heart rate variability (HRV), an established surrogate marker of autonomic nervous system function, was subsequently calculated from beat-to-beat temporal intervals obtained from the PPG signal. HRV metrics were computed via a proprietary algorithm embedded within the device firmware. Importantly, the proprietary algorithm that underpins PPG derived HRV only records during periods where it deems the participant to be asleep, which are derived from heart rate and activity patterns [11]. While the specific parameters and methodology of this algorithm are not publicly disclosed, prior research have demonstrated acceptable agreement with electrocardiogram-derived HRV measures under resting and ambulatory conditions [12, 13]. Moreover, PPG derived heart rate indices have been used in conjunction with advanced modelling for the detection of adverse cardiovascular events [14], demonstrating the applicability of this measurement for clinical physiological measurements [10]. Deidentified participant data were uploaded to a proprietary cloud server and downloaded for analysis at the conclusion of the observational period. HRV data of interest were the root mean square of successive differences between normal heartbeats (RMSSD), and HRV frequency domain indices: low frequency (LF) band (0.04–0.15 Hz), high frequency (HF) band (0.15–0.40 Hz), and their ratio (LF:HF). RMSSD reflects short-term beat-to-beat variation in heart rate and is considered a robust marker of parasympathetic (vagal) activity. While HF has been consistently reported as reflective of cardiac parasympathetic activity [15, 16], there are conflicting viewpoints regarding the interpretation of LF and LF:HF as reflective of sympathetic activity or sympathovagal balance [16–18], Thus, while we report LF:HF ratios for completeness, we acknowledge ongoing debate regarding their interpretation as a surrogate of sympathovagal balance and acknowledge the known limitations [19]. Accordingly, these indices should be interpreted with caution and considered alongside other HRV measures reported.

##### Environmental exposure

Environmental monitoring sensors (SHT45, Sensiron, Switzerland) were utilised to collect temperature (± 0.1 °C accuracy between 5 and 65 °C [41 to 149 °F]) and relative humidity (± 1% accuracy between 15 and 75% at 15 to 55 °C [59 to 131 °F]) in three locations (main living area, secondary living area, main bedroom) of the participant’s home throughout the observational period. The sensor was positioned at 1.1–1.7 m from the floor, on an internal wall, free of likely sources of error (e.g. solar radiation, inhibited airflow, household appliances), and recorded data at 10-min intervals. De-identified recorded data was transferred to a cloud-based storage system over Hypertext Transfer Protocol Secure (HTTPS), ensuring end-to-end encryption of data in transit.

##### Outcome measurement and processing

At the conclusion of the observational period, deidentified participant data were downloaded from respective cloud-based storage systems. Ambient temperature and PPG derived heart rate data were averaged across 5-min epochs within the proprietary algorithm. Heart rate and HRV data were only included within analysis in the event that there were < 5% artefacts in a given period of recording [20]. HRV data were restricted to data recorded between 9 PM and 7 AM to avoid the influence of daytime sleep periods confounding results. HRV indices (RMSSD, LF, HF, LF:HF) were natural log-transformed (ln) prior to statistical analysis to restore normality of distribution. Subsequently, all data were normally distributed.

##### Statistical analysis

The primary outcome of interest was lnRMSSD (ms) derived during nighttime (9 PM–7 AM) sleep. Secondary outcomes included sleep-derived, lnLF, lnHF, and their ratio ln(LF:HF) and heart rate (beats·min^−1^). A ≥ 1.5 standard deviation reduction in lnRMSSD and ≥ 5 beats·min^−1^ change in heart rate from participant normative values were deemed as clinically relevant [21, 22]. Anchored around the World Health Organization recommended upper indoor temperature limit of 26 °C [79 °F], nighttime temperature data were categorised into five ranges: < 24 °C (< 75 °F; reference condition); 24–26 °C (75–79 °F); 26–28 °C (79–82 °F); 28–32 °C (82–90 °F); and > 32 °C (> 90 °F), and included in final analysis if ≥ 1000 participant exposure hours were recorded per range. Secondary analyses were undertaken restricted to individuals not taking medications with strong links to heat sensitivity and impaired thermoregulatory pathways. Data were analysed using linear- and generalised-mixed-effects models where appropriate, accounting for the individual as a random intercept, (two-tailed *α* = 0.05), with R v.4.2.0 (R Foundation).

## Results

Of 79 individuals screened for participation, 47 participated (32 women, median (Q1, Q3) age: 72 (68, 77) years, Table 2). Nighttime bedroom temperature throughout the observational period was 25.9 (24.6, 26.9) °C. Decreased lnRMSSD (*P* < 0.001), lnHF (*P* < 0.001), lnLF (*P* < 0.001) and increased ratio of ln(LF:HF) (*P* < 0.001) and heart rate (*P* < 0.001) were associated with higher nighttime bedroom temperatures. Compared to nighttime bedroom temperatures < 24 °C (75 °F), the odds of experiencing reductions in lnRMSSD were higher when nighttime bedroom temperature was 24–26 °C (75–79 °F; odds ratio: 1.4; 95% confidence interval [1.2, 1.6], *P* < 0.001), 26–28 °C (79–82 °F; 2.0 [1.8, 2.3], *P* < 0.001), and 28–32 °C (82–90 °F; 2.9 [2.5, 3.4], *P* < 0.001). The odds of experiencing increases in ln(LF:HF), heart rate, and decreases in lnHF, lnLF were higher when nighttime bedroom temperature were > 24 °C (75 °F; Table 3), findings that were not influenced by the presence of medication use (Additional File Table 1).

**Table 2:** Physical characteristics of enrolled participants

Variable	All participants (*n* = 47)
Age, *years*	72 (68, 77)
Sex, *No. (%)*	
Females	32 (68%)
Males	15 (32%)
Smoking status^a^*, No. (%)*	
Yes	0 (0%)
No	47 (100%)
Living status, *No. (%)*	
Alone	21 (45%)
Living with partner only	21 (45%)
Living with family/friends	3 (6%)
Other	2 (4%)
Medical conditions*, *No. (%)*	
Cardiac disorder^b^	20 (43%)
Neurological disorder	0 (0%)
Respiratory disorder^c^	15 (32%)
Kidney disorder^d^	2 (4%)
Endocrine disorder^e^	11 (23%)
Other^*f*^	5 (11%)
Using prescribed medications^+^, *No. (%)*	33 (70%)
Anticholinergics	1 (2%)
Beta-blockers	12 (26%)
Antihistamines	8 (17%)
Vasoconstrictors	2 (4%)
Diuretics	0 (0%)
Antiarrhythmics	1 (2%)
Anticoagulants	6 (13%)
Antiepileptics	1 (2%)
Biguanides	5 (11%)
ACE inhibitors	11 (23%)
Antipsychotics	0 (0%)
Antidepressants	3 (6%)
NSAIDs	18 (38%)
Using two or more prescribed medications, *No. (%)*	19 (40%)

Values are median and interquartile range (Q1, Q3) or number of participants (%)

^a^Smoking status determined via participant self-report. Prospective participants were excluded if they were currently smoking

^b^Cardiac disorders: hypertension. No participants reported having medically implanted pacemaker devices

^c^Respiratory disorders: asthma, chronic obstructive pulmonary disorder, non-tubercular mycobacterial lung disease

^d^Kidney disorders: chronic kidney disease

^e^Endocrine disorders: diabetes, hypothyroidism

^f^Other: cancer, osteoporosis, glucose-6-phosphate dehydrogenase deficiency, Von Willebrand disease

^*^All medical conditions determined by participant self-report

^+^Participant prescription medication use determined via self-report. Some participants reported taking medications that have been suggested to increase heat-vulnerability (e.g. antidepressants) or are associated with health conditions known to reduce heat tolerance (e.g. type 2 diabetes, heart disease)

**Table 3:** Heart rate and heart rate variability responses of older adults to increasing bedroom temperature across an entire Australian summer

	Temperature °C [°F]	Data (median [Q1, Q3])	OR [95% CI]	*p*-value
Heart rate (beats·min^−1^)	< 24 [79]	59 [53, 64]	-	-
	24–26 [75–79]	59 [54, 66]	1.2 [1.1–1.3]	0.004
	26–28 [79–82]	59 [54, 65]	1.9 [1.7–2.1]	< 0.001
	28–32 [82–90]	63 [57, 69]	3.9 [3.5–4.4]	< 0.001
lnRMSSD (ms)	< 24 [79]	3.1 [2.7, 3.3]	-	-
	24–26 [75–79]	3.2 [2.9, 3.6]	1.4 [1.2–1.6]	< 0.001
	26–28 [79–82]	3.2 [2.8, 3.6]	2.0 [1.8–2.3]	< 0.001
	28–32 [82–90]	3.1 [2.7, 3.4]	2.9 [2.5–3.4]	< 0.001
lnHF (ms^2^)	< 24 [79]	4.5 [3.7, 5.2]	-	-
	24–26 [75–79]	4.8 [4.0, 5.6]	1.5 [1.3–1.7]	< 0.001
	26–28 [79–82]	4.7 [4.0, 5.6]	2.0 [1.7–2.3]	< 0.001
	28–32 [82–90]	4.5 [3.6, 5.4]	2.4 [2.1–2.9]	< 0.001
lnLF (ms^2^)	< 24 [79]	5.5 [4.9, 6.1]	-	-
	24–26 [75–79]	5.9 [5.1, 6.8]	1.1 [1.0–1.2]	< 0.001
	26–28 [79–82]	6.0 [5.2, 7.0]	1.5 [1.3–1.7]	< 0.001
	28–32 [82–90]	5.8 [4.9, 6.8]	1.8 [1.6–2.1]	< 0.001
Ln(LF:HF)	< 24 [79]	1.1 [0.4, 1.9]	-	-
	24–26 [75–79]	1.1 [0.4, 1.8]	1.2 [1.1–1.3]	< 0.001
	26–28 [79–82]	1.3 [0.5, 1.9]	1.4 [1.2–1.5]	< 0.001
	28–32 [82–90]	1.3 [0.5, 2.0]	1.5 [1.3–1.7]	0.001

*OR* odds ratio, *lnRMSSD* natural logarithm of the root mean square of successive differences between normal heartbeats, *lnHF* natural logarithm of high-frequency (HF) power of the heart rate signal, *lnLF* natural logarithm of low-frequency (LF) power of the heart rate signal, *Ln(LF:HF)* natural logarithm of the ratio of low-frequency to high-frequency power of the heart rate signal. Odds ratios reflect the likelihood of experiencing a clinically relevant decrease in lnRMSSD, lnHF, lnLF, and increase in ln(LF:HF) and heart rate compared to the < 24 °C reference condition. Clinical relevance was defined as an increase in heart rate ≥ 5 beats·min⁻1 and a ≥ 1.5 standard deviation change from each participant’s mean for heart rate variability indices [21]. Estimates were adjusted for repeated measures within individuals. After excluding invalid data (≥ 5% artefacts per hour), 14,179 h from a total of 22,682 recorded hours were included in the final analysis. Exposure hours per temperature category were as follows: < 24 °C (*n* = 2378), 24–26 °C (*n* = 5052), 26–28 °C (*n* = 5705), and 28–32 °C (*n* = 1044). Analysis on the 28–32 °C temperature range was conducted on a reduced sample of participants (*n* = 44) due to three participants failing to experience nighttime bedroom temperatures ≥ 28 °C. Only 3 participant hours were recorded where nighttime bedroom temperature was > 32.0 °C and thus this temperature range was not included within analysis

## Discussion

Exposure to nighttime bedroom temperatures > 24 °C (75 °F) increased the likelihood of clinically relevant alterations in HRV and heart rate in free-living older adults, with more pronounced effects observed once nighttime bedroom temperatures exceeded 26 °C (79 °F). The observed changes in HRV markers suggest a shift towards sympathetic dominance in autonomic nervous system activity and greater physiological stress as nighttime bedroom temperatures increase. These findings have significant implications for the development of data-driven recommendations for the maintenance of nighttime indoor temperature limits, particularly within a rapidly warming world.

Given epidemiological evidence demonstrating adverse health outcomes associated with hot-nights [1, 5] and the absence of established guidelines for safe indoor nighttime temperatures [23], our findings highlight the importance of maintaining nighttime bedroom temperatures ≤ 24 °C (75 °F) whenever possible. While the impacts of the observed changes in heart rate and HRV on heat-related health outcomes remains uncertain, our findings expand on previously published literature that shows increased daily temperatures (city-wide estimates) are associated with increased nighttime blood pressure responses in individuals with hypertension [24, 25]. Together, the observed autonomic dysregulation in the current study, alongside alterations in blood pressure previously observed [24, 25], may represent early physiological markers of reduced nocturnal recovery, which over time could contribute to increased cardiovascular strain and risk, particularly in older adults and those with pre-existing health conditions. Such effects may plausibly exacerbate pathways leading to target organ damage and acute cardiovascular events.

Our findings provide real-world physiological evidence that complements the established epidemiological associations between hot nights and adverse health outcomes. By directly capturing autonomic responses under free-living conditions, this study addresses a critical gap in the literature and strengthens the case for incorporating nighttime temperature limits into public health guidance. Given climate-projections indicate that hot nights will become more frequent and severe and will account for a growing proportion of heat-related deaths, proactive measures to maintain safe nighttime indoor environments are warranted. These include the development of indoor monitoring systems and individualised early warning systems [26, 27] which act as a real-time safeguard by identifying unsafe thermal conditions, alerting at-risk individuals, and prompting timely behavioural or environmental countermeasures to prevent physiological strain and adverse health outcomes. Future longitudinal research should determine whether the autonomic alterations observed here translate into measurable increases in cardiovascular morbidity and mortality, thereby refining threshold values for effective nighttime heat mitigation strategies.

In many countries, heatwaves are typically characterised solely by elevated daytime temperatures, with little consideration given to elevated nighttime temperatures [28]. However, our findings reinforce the importance of quantifying not just extreme daytime highs but also elevated nighttime temperatures. It is likely that the cumulative influence of successive hot nights compounds the physiological burden imposed by hot days, a notion supported by epidemiological evidence [29]. This may be resultant from consecutive periods of elevated daytime and nighttime temperatures limiting the capacity for cardiovascular and autonomic recovery, resulting in a sustained state of sympathetic dominance, impaired parasympathetic activity, and elevated cardiovascular strain. In this context, hot nights not only represent an independent risk factor but may also exacerbate the residual effects of daytime heat exposure, creating a compounding cycle of physiological stress. Over time, this may accelerate the progression of heat-related morbidity, particularly in older adults and those with pre-existing cardiovascular disease, underscoring the importance of interventions that target both daytime and nighttime thermal exposures.

While we present a robust analysis of the autonomic responses to increasing nighttime bedroom temperatures, some limitations of the study must be considered when interpreting our findings. First, our participant cohort was a small, homogeneous sample of older adults residing in a sub-tropical climate, whom had likely garnered some level of heat acclimatization. Thus, it is unclear if similar results would be observed for those residing in hot-dry and arid climates, and non-acclimatized individuals. Second, the use of wearable-grade devices was a necessary constraint of this observational study. While these devices enable large-scale, unobtrusive, and continuous monitoring under real-world conditions, they inherently rely on proprietary algorithms to derive HRV metrics from PPG-derived inter-beat intervals, during periods where the end-user is deemed to be asleep. We acknowledge that relying on this proprietary detection of periods of sleep (or wakefulness) has the potential to influence results. Finally, it was not possible to determine participant use of active and passive interventions that may have increased the potential for evaporative heat loss and subsequent autonomic responses without altering bedroom temperature (i.e. fans [22, 30], natural ventilation). Despite these limitations, the capacity for continuous overnight monitoring allowed for high-resolution assessment of nocturnal autonomic function under real-world environmental conditions, overcoming the logistical and ethical challenges of invasive or laboratory-based investigations, or laboratory-grade monitoring devices.

## Conclusions

Our findings provide novel in-home physiological evidence linking elevated nighttime temperatures with autonomic disruption, reinforcing epidemiological associations between hot nights and adverse health outcomes. We herein note that while data were collected during the nocturnal period, this investigation was not designed as a sleep study per se; rather, nighttime monitoring was employed to minimise behavioural impacts (e.g. physical activity) on HRV, therefore isolating the impact of increasing bedroom temperatures on autonomic and cardiovascular responses. Future research should explore interventions that mitigate nighttime heat exposure and determine whether improving nocturnal thermal environments translates to reduced cardiovascular strain and downstream health outcomes, thereby informing the development of public health guidelines that incorporate explicit nighttime temperature thresholds. In addition, dedicated sleep-focused studies that incorporate detailed circadian HRV indices and sleep architecture are warranted to further elucidate the mechanisms by which hot nights disrupt nocturnal recovery and contribute to long-term cardiovascular risk.

## Supplementary Information

Additional file 1: Table 1 Likelihood of experiencing clinically relevant alterations in heart rate and heart rate variability responses of older adults to increasing bedroom temperature across an entire Australian summer. Data presented as individuals taking medications with links to heat sensitivity and impaired thermoregulation (*n* = 26) and restricted to individuals not taking these medications (*n* = 21). Table 2 Descriptive analysis of hourly nighttime bedroom temperature per participant across the observational period.

## Data Availability

Deidentified participant data will be made available upon reasonable request to the corresponding author. All data requests should include a detailed re-analysis plan and will require a signed access agreement.

## Abbreviations

Cfa: Köppen climate classification for humid subtropical climate
°C: Degrees Celsius
°F: Degrees Fahrenheit
ECG: Electrocardiogram
HF: High frequency (band; 0.15–0.40 Hz)
HR: Heart rate
HRV: Heart rate variability
HTTPS: Hypertext Transfer Protocol Secure
LF: Low frequency (band; 0.04–0.15 Hz)
LF:HF: Ratio of low frequency to high frequency components of HRV
ln: Natural logarithm
lnHF: Natural logarithm of high frequency heart rate variability
lnLF: Natural logarithm of low frequency heart rate variability
lnRMSSD: Natural logarithm of the root mean square of successive differences between normal heartbeats
ms: Milliseconds
PPG: Photoplethysmography
Q1, Q3: First and third quartiles
RMSSD: Root mean square of successive differences between normal heartbeats
WHO: World Health Organization
