# Supplementary material for: Effect of nighttime bedroom temperature on heart rate variability in older adults: an observational study
Source: BMC Med. 2025 Dec 29;23:703. doi: 10.1186/s12916-025-04513-0 (PMC12751657; doi:10.1186/s12916-025-04513-0)
Supplement: Supplementary file 1 — Additional file 1: Table 1 Likelihood of experiencing clinically relevant alterations in heart rate and heart rate variability responses of older adults to increasing bedroom temperature across an entire Australian summer. Data presented as individuals taking medications with links to heat sensitivity and impaired thermoregulation (n = 26) and restricted to individuals not taking these medications (n = 21). Table 2 Descriptive analysis of hourly nighttime bedroom temperature per participant across the observational period. [file 12916_2025_4513_MOESM1_ESM.docx]

| **Table 1.** Likelihood of experiencing clinically relevant alterations in heart rate and heart rate variability responses of older adults to increasing bedroom temperature across an entire Australian summer. Data presented as individuals taking medications with links to heat sensitivity and impaired thermoregulation (n=26) and restricted to individuals not taking these medications (n=21). | | | | | | |  |
| --- | --- | --- | --- | --- | --- | --- | --- |
|  | Temperature (°C) | OR [95% CI] (n=26) | | OR [95% CI] (n=21) | | |  |
| Heart Rate (beats·min^-1^) | <24 | - | | - |  | |  |
|  | 24-26 | 1.5 [1.3 - 1.7] | | 1.1 [1.0 - 1.3] |  | |  |
|  | 26-28 | 2.5 [2.1 - 3.0] | | 1.7 [1.5 - 1.9] |  | |  |
|  | 28-32 | 4.8 [3.8 - 6.0] | | 3.6 [3.1 - 4.1] |  | |  |
| lnRMSSD (ms) | <24 | - | - | | |  | |
|  | 24-26 | 1.6 [1.3 - 1.9] | 1.2 [1.0 - 2.4] | | |  | |
|  | 26-28 | 2.1 [1.7 - 2.6] | 1.8 [1.5 - 2.2] | | |  | |
|  | 28-32 | 3.8 [3.0 - 5.0] | 2.4 [2.0 - 3.0] | | |  | |
| lnHF (ms^2^) | <24 | - | - | | |  | |
|  | 24-26 | 1.8 [1.5 – 2.1] | 1.3 [1.1 - 1.6] | | |  | |
|  | 26-28 | 2.2 [1.8 - 2.8] | 1.8 [1.6 - 2.2] | | |  | |
|  | 28-32 | 3.3 [2.5 - 4.3] | 2.1 [1.7 - 2.6] | | |  | |
| lnLF (ms^2^) | <24 | - | - | | |  | |
|  | 24-26 | 1.1 [0.9 - 1.4] | 1.1 [1.0 - 1.3] | | |  | |
|  | 26-28 | 1.5 [1.3 - 1.9] | 1.5 [1.3 - 1.7] | | |  | |
|  | 28-32 | 1.9 [1.4 - 2.6] | 1.8 [1.5 - 2.1] | | |  | |
| ln(LF:HF) | <24 | - | - | | |  | |
|  | 24-26 | 1.1 [0.9 - 1.3] | 1.2 [1.1 - 1.4] | | |  | |
|  | 26-28 | 1.4 [1.1 - 1.6] | 1.4 [1.2 - 1.6] | | |  | |
|  | 28-32 | 1.7 [1.3 - 2.2] | 1.5 [1.2 – 1.7] | | |  | |
| OR: odds ratio. lnRMSSD: natural logarithm of the root mean square of successive differences between normal heartbeats. lnHF: natural logarithm of the high-frequency power spectral density derived from beat-to-beat temporal intervals obtained from the PPG signal. lnLF: natural logarithm of the low-frequency power spectral density derived from beat-to-beat temporal intervals obtained from the PPG signal. ln(LF/HF): natural logarithm of the ratio between low-frequency and high-frequency power spectral densities. Odds ratios reflect the likelihood of experiencing a clinically relevant decrease in lnRMSSD, lnHF, lnLF, and increase in ln(LF:HF) and heart rate compared to the 24 °C reference condition.  Clinical relevance was defined as an increase in heart rate ≥5 beats·min^-1^ and a ≥1.5 standard deviation change from each participant's mean for heart rate variability indices [15]. Estimates were adjusted for repeated measures within individuals. Heat sensitivity medications defined as anticholinergics, beta-blockers, antihistamines, vasoconstrictors, diuretics, antipsychotics and antidepressants. | | | | | |  | |

| **Table 2.** Descriptive analysis of hourly nighttime bedroom temperature per participant across the observational period | | | | | | |  |
| --- | --- | --- | --- | --- | --- | --- | --- |
|  |  | **Percentiles** | | |  |  |  |
| **userId** | **Median** | **25th** | **75th** | **95th** | **Maximum** |  |  |
| **1** | 26.0 | 25.3 | 26.8 | 27.9 | 31.4 |  |  |
| **5** | 25.1 | 24.1 | 25.9 | 27.2 | 30.4 |  |  |
| **9** | 26.6 | 25.4 | 27.9 | 29.3 | 31.1 |  |  |
| **13** | 26.4 | 25.3 | 27.7 | 29.3 | 31.5 |  |  |
| **17** | 26.6 | 25.6 | 27.5 | 28.9 | 32.0 |  |  |
| **21** | 26.2 | 25.2 | 27.3 | 29.2 | 32.1 |  |  |
| **25** | 24.8 | 23.8 | 25.5 | 26.5 | 27.6 |  |  |
| **29** | 26.6 | 25.8 | 27.3 | 28.4 | 31.0 |  |  |
| **33** | 25.6 | 24.8 | 26.3 | 26.9 | 28.0 |  |  |
| **37** | 26.6 | 25.5 | 27.9 | 29.8 | 33.6 |  |  |
| **41** | 25.7 | 24.6 | 26.5 | 28.0 | 29.6 |  |  |
| **45** | 21.9 | 21.2 | 22.9 | 26.4 | 30.6 |  |  |
| **53** | 26.7 | 25.8 | 27.4 | 28.2 | 29.3 |  |  |
| **57** | 25.7 | 24.7 | 26.6 | 27.6 | 28.9 |  |  |
| **61** | 26.9 | 25.9 | 27.9 | 29.0 | 30.6 |  |  |
| **65** | 25.6 | 24.3 | 26.6 | 27.9 | 29.8 |  |  |
| **73** | 24.1 | 23.5 | 25.4 | 27.0 | 28.7 |  |  |
| **81** | 25.8 | 24.8 | 26.7 | 27.7 | 30.4 |  |  |
| **85** | 27.4 | 26.7 | 28.0 | 28.8 | 30.0 |  |  |
| **93** | 26.8 | 25.8 | 27.6 | 28.8 | 30.0 |  |  |
| **97** | 25.2 | 23.7 | 26.4 | 28.0 | 29.7 |  |  |
| **101** | 25.4 | 24.5 | 26.6 | 28.3 | 30.1 |  |  |
| **105** | 26.4 | 25.5 | 27.4 | 29.1 | 31.5 |  |  |
| **109** | 26.2 | 25.4 | 26.9 | 27.8 | 29.8 |  |  |
| **113** | 24.6 | 23.7 | 25.4 | 26.7 | 29.1 |  |  |
| **125** | 26.1 | 25.0 | 26.8 | 27.8 | 29.6 |  |  |
| **133** | 27.3 | 26.5 | 28.1 | 29.4 | 31.1 |  |  |
| **137** | 22.3 | 21.3 | 26.0 | 28.3 | 31.3 |  |  |
| **145** | 26.4 | 25.8 | 27.1 | 27.8 | 29.6 |  |  |
| **149** | 24.7 | 23.5 | 26.4 | 28.5 | 31.1 |  |  |
| **161** | 27.1 | 26.5 | 27.6 | 28.4 | 29.6 |  |  |
| **165** | 26.3 | 25.3 | 27.2 | 28.3 | 29.4 |  |  |
| **169** | 26.2 | 25.0 | 27.1 | 28.0 | 29.7 |  |  |
| **173** | 26.1 | 25.2 | 27.1 | 28.6 | 32.1 |  |  |
| **177** | 25.9 | 24.6 | 27.1 | 29.1 | 32.6 |  |  |
| **181** | 24.8 | 23.8 | 25.6 | 26.7 | 27.5 |  |  |
| **189** | 25.5 | 23.6 | 26.7 | 27.9 | 29.4 |  |  |
| **201** | 24.9 | 23.6 | 26.2 | 28.0 | 32.9 |  |  |
| **205** | 24.9 | 23.3 | 25.7 | 27.5 | 30.6 |  |  |
| **209** | 26.7 | 25.7 | 27.8 | 29.5 | 32.8 |  |  |
| **213** | 24.2 | 23.3 | 25.0 | 26.3 | 29.6 |  |  |
| **221** | 22.8 | 21.9 | 23.6 | 24.8 | 26.0 |  |  |
| **229** | 26.9 | 26.1 | 27.8 | 29.1 | 31.6 |  |  |
| **249** | 26.7 | 26.0 | 27.2 | 28.1 | 29.7 |  |  |
| **253** | 26.7 | 25.6 | 27.8 | 29.3 | 31.9 |  |  |
| **265** | 26.0 | 24.9 | 27.0 | 28.3 | 29.1 |  |  |
| **273** | 26.0 | 25.1 | 27.0 | 28.2 | 30.3 |  |  |
| **277** | 25.8 | 25.0 | 26.6 | 27.5 | 28.8 |  |  |
